# Acceleration of electrons in the plasma wakefield of a proton bunch

**DOI:** 10.1038/s41586-018-0485-4

**Published:** 2018-08-29

**Authors:** E. Adli, A. Ahuja, O. Apsimon, R. Apsimon, A.-M. Bachmann, D. Barrientos, F. Batsch, J. Bauche, V. K. Berglyd Olsen, M. Bernardini, T. Bohl, C. Bracco, F. Braunmüller, G. Burt, B. Buttenschön, A. Caldwell, M. Cascella, J. Chappell, E. Chevallay, M. Chung, D. Cooke, H. Damerau, L. Deacon, L. H. Deubner, A. Dexter, S. Doebert, J. Farmer, V. N. Fedosseev, R. Fiorito, R. A. Fonseca, F. Friebel, L. Garolfi, S. Gessner, I. Gorgisyan, A. A. Gorn, E. Granados, O. Grulke, E. Gschwendtner, J. Hansen, A. Helm, J. R. Henderson, M. Hüther, M. Ibison, L. Jensen, S. Jolly, F. Keeble, S.-Y. Kim, F. Kraus, Y. Li, S. Liu, N. Lopes, K. V. Lotov, L. Maricalva Brun, M. Martyanov, S. Mazzoni, D. Medina Godoy, V. A. Minakov, J. Mitchell, J. C. Molendijk, J. T. Moody, M. Moreira, P. Muggli, E. Öz, C. Pasquino, A. Pardons, F. Peña Asmus, K. Pepitone, A. Perera, A. Petrenko, S. Pitman, A. Pukhov, S. Rey, K. Rieger, H. Ruhl, J. S. Schmidt, I. A. Shalimova, P. Sherwood, L. O. Silva, L. Soby, A. P. Sosedkin, R. Speroni, R. I. Spitsyn, P. V. Tuev, M. Turner, F. Velotti, L. Verra, V. A. Verzilov, J. Vieira, C. P. Welsch, B. Williamson, M. Wing, B. Woolley, G. Xia

**Affiliations:** 1 University of Oslo, Oslo, Norway; 2 CERN, Geneva, Switzerland; 3 University of Manchester, Manchester, UK; 4 Cockcroft Institute, Daresbury, UK; 5 Lancaster University, Lancaster, UK; 6 Max Planck Institute for Physics, Munich, Germany; 7 Technical University Munich, Munich, Germany; 8 Max Planck Institute for Plasma Physics, Greifswald, Germany; 9 UCL, London, UK; 10 UNIST, Ulsan, South Korea; 11 Philipps-Universität Marburg, Marburg, Germany; 12 Heinrich-Heine-University of Düsseldorf, Düsseldorf, Germany; 13 University of Liverpool, Liverpool, UK; 14 ISCTE—Instituto Universitéario de Lisboa, Lisbon, Portugal; 15 Budker Institute of Nuclear Physics SB RAS, Novosibirsk, Russia; 16 Novosibirsk State University, Novosibirsk, Russia; 17 Technical University of Denmark, Lyngby, Denmark; 18 GoLP/Instituto de Plasmas e Fusão Nuclear, Instituto Superior Técnico, Universidade de Lisboa, Lisbon, Portugal; 19 TRIUMF, Vancouver, British Columbia Canada; 20 Ludwig-Maximilians-Universität, Munich, Germany; 21 Institute of Computational Mathematics and Mathematical Geophysics SB RAS, Novosibirsk, Russia; 22 University of Milan, Milan, Italy

**Keywords:** Proton Bunch, Plasma Wakefield, Microbunches, Electron Bunch, Background Data Samples, Plasma-based accelerators, Experimental particle physics

## Abstract

High-energy particle accelerators have been crucial in providing a deeper understanding of fundamental particles and the forces that govern their interactions. To increase the energy of the particles or to reduce the size of the accelerator, new acceleration schemes need to be developed. Plasma wakefield acceleration^1–5^, in which the electrons in a plasma are excited, leading to strong electric fields (so called ‘wakefields’), is one such promising acceleration technique. Experiments have shown that an intense laser pulse^6–9^ or electron bunch^10,11^ traversing a plasma can drive electric fields of tens of gigavolts per metre and above—well beyond those achieved in conventional radio-frequency accelerators (about 0.1 gigavolt per metre). However, the low stored energy of laser pulses and electron bunches means that multiple acceleration stages are needed to reach very high particle energies^5,12^. The use of proton bunches is compelling because they have the potential to drive wakefields and to accelerate electrons to high energy in a single acceleration stage^13^. Long, thin proton bunches can be used because they undergo a process called self-modulation^14–16^, a particle–plasma interaction that splits the bunch longitudinally into a series of high-density microbunches, which then act resonantly to create large wakefields. The Advanced Wakefield (AWAKE) experiment at CERN^17–19^ uses high-intensity proton bunches—in which each proton has an energy of 400 gigaelectronvolts, resulting in a total bunch energy of 19 kilojoules—to drive a wakefield in a ten-metre-long plasma. Electron bunches are then injected into this wakefield. Here we present measurements of electrons accelerated up to two gigaelectronvolts at the AWAKE experiment, in a demonstration of proton-driven plasma wakefield acceleration. Measurements were conducted under various plasma conditions and the acceleration was found to be consistent and reliable. The potential for this scheme to produce very high-energy electron bunches in a single accelerating stage^20^ means that our results are an important step towards the development of future high-energy particle accelerators^21,22^.

## Abstract (web summary)

Electron acceleration to very high energies is achieved in a single step by injecting electrons into a ‘wake’ of charge created in a 10-metre-long plasma by speeding long proton bunches.

## Main

**Fig. 1:**
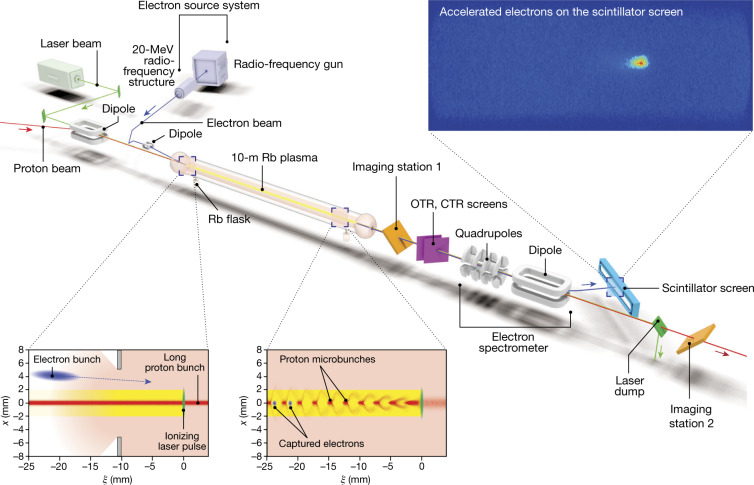
Layout of the AWAKE experiment. The proton bunch and laser pulse propagate from left to right across the image, through a 10-m column of rubidium (Rb) vapour. This laser pulse (green, bottom images) singly ionizes the rubidium to form a plasma (yellow), which then interacts with the proton bunch (red, bottom left image). This interaction modulates the long proton bunch into a series of microbunches (bottom right image), which drive a strong wakefield in the plasma. These microbunches are millimetre-scale in the longitudinal direction (*ξ*) and submillimetre-scale in the transverse (*x*) direction. The self-modulation of the proton bunch is measured in imaging stations 1 and 2 and the optical and coherent transition radiation (OTR, CTR) diagnostics. The rubidium (pink) is supplied by two flasks at each end of the vapour source. The density is controlled by changing the temperature in these flasks and a gradient may be introduced by changing their relative temperature. Electrons (blue), generated using a radio-frequency source, propagate a short distance behind the laser pulse and are injected into the wakefield by crossing at an angle. Some of these electrons are captured in the wakefield and accelerated to high energies. The accelerated electron bunches are focused and separated from the protons by the quadrupoles and dipole magnet of the spectrometer (grey, right). These electrons interact with a scintillating screen, creating a bright intensity spot (top right image), allowing them to be imaged and their energy inferred from their position.

The layout of the AWAKE experiment is shown in Fig. 1. A proton bunch from CERN’s Super Proton Synchrotron (SPS) accelerator co-propagates with a laser pulse (green), which creates a plasma (yellow) in a column of rubidium vapour (pink) and seeds the modulation of the proton bunch into microbunches (Fig. 1; red, bottom images). The protons have an energy of 400 GeV and the root-mean-square (r.m.s.) bunch length is 6–8 cm^18^. The bunch is focused to a transverse size of approximately 200 μm (r.m.s.) at the entrance of the vapour source, with the bunch population varying shot-to-shot in the range *N*_p_ ≈ (2.5–3.1) × 10^11^ protons per bunch. Proton extraction occurs every 15–30 s. The laser pulse used to singly ionize the rubidium in the vapour source^23,24^ is 120 fs long with a central wavelength of 780 nm and a maximum energy of 450 mJ^25^. The pulse is focused to a waist of approximately 1 mm (full-width at half-maximum, FWHM) inside the rubidium vapour source, five times the transverse size of the proton bunch. The rubidium vapour source (Fig. 1; centre) has a length of 10 m and diameter of 4 cm, with rubidium flasks at each end. The rubidium vapour density and hence the plasma density *n*_pe_ can be varied in the range 10^14^–10^15^ cm^−3^ by heating the rubidium flasks to temperatures of 160–210 °C. This density range corresponds to a plasma wavelength of 1.1–3.3 mm, as detailed in Methods. A gradient in the plasma density can be introduced by heating the rubidium flasks to different temperatures. Heating the downstream (Fig. 1; right side) flask to a higher temperature than the upstream (left side) flask creates a positive density gradient, and vice versa. Gradients in plasma density have been shown in simulation to produce large increases in the maximum energy attainable by the injected electrons^26^. The effect of density gradients here is different from that for short drivers^27^. In addition to keeping the wake travelling at the speed of light at the witness position, the gradient prevents destruction of the bunches at the final stage of self-modulation^28^, thus increasing the wakefield amplitude at the downstream part of the plasma cell. The rubidium vapour density is monitored constantly by an interferometer-based diagnostic^29^.

The self-modulation of the proton bunch into microbunches (Fig. 1; red, bottom right image) is measured using optical and coherent transition radiation diagnostics (Fig. 1; purple)^30^. However, these diagnostics have a destructive effect on the accelerated electron bunch and cannot be used during electron acceleration experiments. The second beam-imaging station (Fig. 1; orange, right) is used instead, providing an indirect measurement of the self-modulation by measuring the transversely defocused protons^31^. These protons are expelled from the central propagation axis by transverse electric fields that are present only when the proton bunch undergoes modulation in the plasma.

Electron bunches with a charge of 656 ± 14 pC (where the uncertainty is the r.m.s.) are produced and accelerated to 18.84 ± 0.05 MeV (where the uncertainty is the standard error of the mean) in a radio-frequency structure upstream of the vapour source^32^. These electrons are then transported along a beam line before being injected into the vapour source. Magnets along the beam line are used to control the injection angle and focal point of the electrons. For the results presented here, the electrons enter the plasma with a small vertical offset with respect to the proton bunch and a 200-ps delay with respect to the ionizing laser pulse (Fig. 1, bottom left). The beams cross approximately 2 m into the vapour source at a crossing angle of 1.2–2 mrad. Simulations show that electrons are captured in larger numbers and accelerated to higher energies when injected off-axis rather than collinearly with the proton bunch^17^. The normalized emittance of the witness electron beam at injection is approximately 11–14 mm mrad and its focal point is close to the entrance of the vapour source. The delay of 200 ps corresponds to approximately 25 proton microbunches resonantly driving the wakefield at *n*_pe_ = 2 × 10^14^ cm^−3^ and 50 microbunches at *n*_pe_ = 7 × 10^14^ cm^−3^.

A magnetic electron spectrometer (Fig. 1, right) enables measurement of the accelerated electron bunch^33^. Two quadrupole magnets are located 4.48 m and 4.98 m downstream of the exit iris of the vapour source and focus the witness beam vertically and horizontally, respectively, to more easily identify a signal. These are followed by a 1-m-long C-shaped electromagnetic dipole with a maximum magnetic field of approximately 1.4 T. A large triangular vacuum chamber sits in the cavity of the dipole. This chamber is designed to keep accelerated electron bunches under vacuum while the magnetic field of the dipole induces an energy-dependent horizontal deflection in the bunch. Electrons within a specific energy range then exit this vacuum chamber through a 2-mm-thick aluminium window and are incident on a 0.5-mm-thick gadolinium oxysulfide (Gd_2_O_2_S:Tb) scintillator screen (Fig. 1; blue, right) attached to the exterior surface of the vacuum chamber. The proton bunch is not greatly affected by the spectrometer magnets, owing to its high momentum, and continues to the beam dump. The scintillating screen is 997 mm wide and 62 mm high with semi-circular ends. Light emitted from the scintillator screen is transported over a distance of 17 m via three highly reflective optical-grade mirrors to an intensified charge-coupled device (CCD) camera fitted with a lens with a focal length of 400 mm. The camera and the final mirror of this optical line are housed in a dark room, which reduces ambient light incident on the camera to negligible values.

The energy of the accelerated electrons is inferred from their horizontal position in the plane of the scintillator. The relationship between this position and the energy of the electron is dependent on the strength of the dipole, which can be varied from approximately 0.1 T to 1.4 T. This position–energy relationship has been simulated using the Beam Delivery Simulation (BDSIM) code^34^. The simulation tracks electrons of various energies through the spectrometer using measured and simulated magnetic-field maps for the spectrometer dipole, as well as the relevant distances between components. The accuracy of the magnetic-field maps, the precision of the distance measurements and the 1.5-mm resolution of the optical system lead to an energy uncertainty of approximately 2%. The overall uncertainty, however, is dominated by the emittance of the accelerated electrons, and can be larger than 10%. The use of the focusing quadrupoles limits this uncertainty to approximately 5% for electrons near to the focused energy.

Owing to the difficulty of propagating an electron beam of well-known intensity to the spectrometer at AWAKE, the charge response of the scintillator is calculated using data acquired at CERN’s Linear Electron Accelerator for Research (CLEAR) facility. This calibration is performed by placing the scintillator and vacuum window next to a beam charge monitor on the CLEAR beam line and measuring the scintillator signal. The response of the scintillator is found to depend linearly on charge over the range 1–50 pC. The response is also found to be independent of position and of energies in the range 100–180 MeV, to within the measurement uncertainty. This charge response is then recalculated for the optical system of the spectrometer at AWAKE by imaging a well-known light source at both locations. A response of (6.9 ± 2.1) × 10^6^ CCD counts per incident picocoulomb of charge, given the acquisition settings used at AWAKE, is determined. The large 1*σ* uncertainty is due to different triggering conditions at CLEAR and AWAKE and systematic uncertainties in the calibration results.

Reliable acceleration of electrons relies on reproducible self-modulation of the proton beam. As well as the observation of the transverse expansion of the proton bunch, the optical and coherent transition radiation diagnostics showed clear microbunching of the beam. The proton microbunches were observed to be separated by the plasma wavelength (inferred from the measured rubidium vapour density, see Methods) for all parameter ranges investigated; they were also reproducible and stable in phase relative to the seeding. The detailed study of the self-modulation process will be the subject of separate AWAKE publications.

**Fig. 2:**
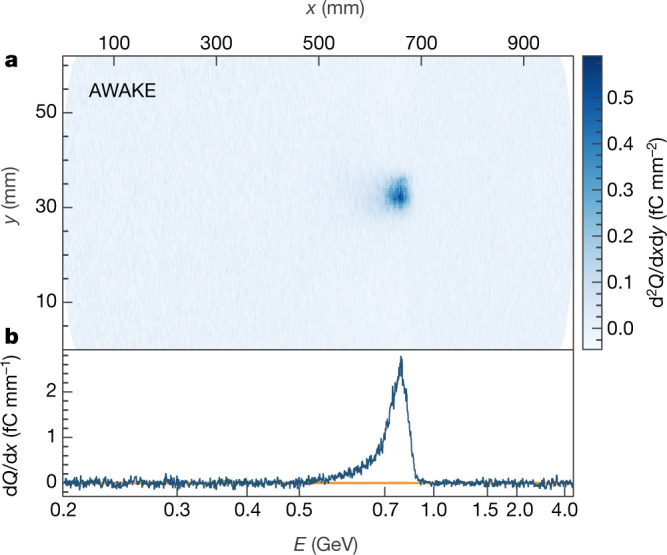
Signal of accelerated electrons. **a**, An image of the scintillator (with horizontal distance *x* and vertical distance *y*) with background subtraction and geometric corrections applied is shown, with an electron signal clearly visible. The intensity of the image is given in charge *Q* per unit area (d^2^*Q*/d*x*d*y*), calculated using the central value from the calibration of the scintillator. **b**, A projection of the image in **a** is obtained by integrating vertically over the charge observed in the central region of the image. A 1*σ* uncertainty band from the background subtraction is shown in orange around zero. Both the image (**a**) and the projection (**b**) are binned in space, as shown on the top axis, but the central value from the position–energy conversion is indicated at various points on the bottom axis. The electron signal is clearly visible above the noise, with a peak intensity at an energy of *E* ≈ 800 MeV.

The data presented here were collected in May 2018. In Fig. 2a we show an image of the scintillator from an electron acceleration event at a plasma density of 1.8 × 10^14^ cm^−3^, with a measured density difference of +5.3% ± 0.3% over 10 m in the direction of propagation of the proton bunch. This image has been background-subtracted and corrected for vignetting and electron-angle effects (Methods). The quadrupoles of the spectrometer were focusing at an energy of approximately 700 MeV during this event, creating a substantial reduction in the vertical spread of the beam. In Fig. 2b we show a projection obtained by integrating over a central region of the scintillator. A 1*σ* uncertainty band, which comes from the background subtraction, is shown around zero. The peak in this figure has a high signal-to-noise ratio, which provides clear evidence of accelerated electrons. In both the image and the projection, the charge density is calculated using the central value of 6.9 × 10^6^ CCD counts per picocoulomb. The asymmetric shape of the peak is due to the nonlinear position–energy relationship induced in the electron bunch by the magnetic field; when re-binned in energy, the signal peak is approximately Gaussian. Accounting for the systematic uncertainties described earlier, the observed peak has a mean of 800 ± 40 MeV, a FWHM of 137.3 ± 13.7 MeV and a total charge of 0.249 ± 0.074 pC. The amount of charge captured is expected to increase considerably^17^ as the emittance of the injected electron bunch is reduced and its geometric overlap with the wakefield is improved.

**Fig. 3:**
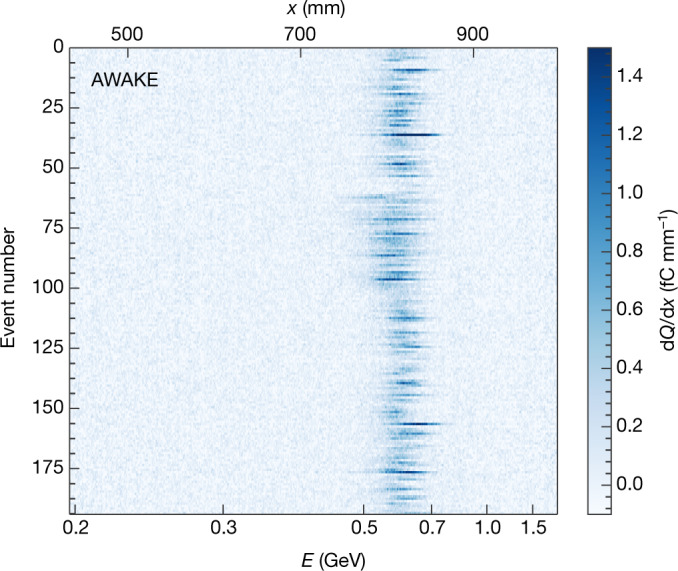
Background-subtracted projections of consecutive electron-injection events. Each projection (event) is a vertical integration over the central region of a background-subtracted spectrometer camera image. Brighter colours indicate regions of high charge density d*Q*/d*x*, corresponding to accelerated electrons. The quadrupoles of the spectrometer were varied to focus at energies of 460–620 MeV over the duration of the dataset. No other parameters were varied deliberately. The consistent peak around energy *E* ≈ 600 MeV demonstrates the stability and reliability of the electron acceleration.

The stability and reliability of the electron acceleration is evidenced by Fig. 3, which shows projections from many consecutive electron-injection events. Each row in this plot is the background-subtracted projection from a single event, with the colour representing the signal intensity. The events correspond to a 2-h running period during which the quadrupoles were varied to focus over a range of approximately 460–620 MeV. Other parameters, such as the proton-bunch population, were not deliberately changed but vary naturally on a shot-to-shot basis. Despite the quadrupole scan and the natural fluctuations in the beam parameters, the plot still shows consistent and reproducible acceleration of electron bunches to approximately 600 MeV. The plasma density for these events is 1.8 × 10^14^ cm^−3^, with no density gradient. This lack of gradient is the cause of the difference in energy between the event in Fig. 2 and the events in Fig. 3.

**Fig. 4:**
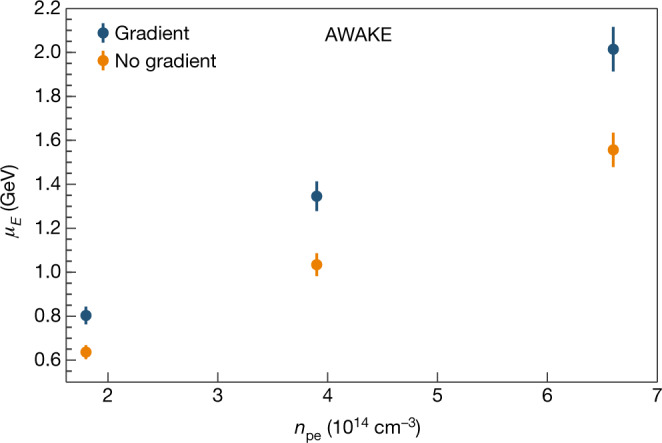
Measurement of the highest peak energies *μ*_*E*_ achieved at different plasma densities *n*_pe_, with and without a gradient in the plasma density. The error bars arise from the position–energy conversion. The gradients chosen are those that were observed to maximize the energy gain. Acceleration to 2.0 ± 0.1 GeV is achieved with a plasma density of 6.6 × 10^14^ cm^−3^ with a density difference of +2.2% ± 0.1% over 10 m.

The energy gain achievable by introducing a more optimal gradient is demonstrated in Fig. 4, which shows the peak energy achieved at different plasma densities with and without a gradient. The density gradients chosen are those that are observed to maximize the peak energy for a given plasma density. At 1.8 × 10^14^ cm^−3^ the density difference was approximately +5.3% ± 0.3% over 10 m, whereas at 3.9 × 10^14^ cm^−3^ and 6.6 × 10^14^ cm^−3^ it fell to +2.5% ± 0.3% and +2.2% ± 0.1%, respectively. Given the precise control of the longitudinal plasma density, small density gradients can have a substantial effect on the acceleration because the electrons are injected tens of microbunches behind the ionizing laser pulse^26^. The charge of the observed electron bunches decreases at higher plasma densities, owing in part to the smaller transverse size of the wakefield. In addition, the quadrupoles of the spectrometer have a maximum focusing energy of 1.3 GeV, which makes bunches accelerated to higher energies than this harder to detect above the background noise.

The energies shown in Fig. 4 are determined by binning the pixel data in energy and fitting a Gaussian over the electron signal region; the peak energy *μ*_*E*_ is the mean of this Gaussian. The observed energy spread of each bunch is determined by the width of this Gaussian and is approximately 10% of the peak energy. The peak energy increases with density, reaching 2.0 ± 0.1 GeV for *n*_pe_ = 6.6 × 10^14^ cm^−3^ in the presence of a density gradient, at which point the charge capture is much lower. The energies of the accelerated electrons are within the range of values originally predicted by particle-in-cell and fluid code simulations of the AWAKE experiment^17,18,26^. Future data-collection runs will address the effect of the electron-bunch delay, injection angle and other parameters on the accelerated energy and charge capture. These studies will help to determine what sets the limit on the energy gain.

In summary, we have demonstrated proton-driven plasma wakefield acceleration. The strong electric fields, generated by a series of proton microbunches, were sampled with a bunch of electrons. These electrons were accelerated up to 2 GeV in approximately 10 m of plasma and measured using a magnetic spectrometer. This technique has the potential to accelerate electrons to the teraelectronvolt scale in a single accelerating stage. Although still in the early stages of its programme, the AWAKE experiment is an important step towards realizing new high-energy particle physics experiments.

## Methods

### Plasma generation

A CentAurus Ti:sapphire laser system is used to ionize the rubidium in the vapour source. The rubidium is confined by expansion chambers at the ends of the source with 10-mm-diameter irises through which rubidium flows constantly and condensates on the expansion walls. By the relation *λ*_pe_ = 2π*c*[*ε*_0_*m*_e_/(*n*_pe_*e*^2^)]^1/2^, where *c* is the speed of light, *ε*_0_ is the permittivity of free space, *m*_e_ is the electron mass and *e* is the electron charge, the available density range of *n*_pe_ = 10^14^–10^15^ cm^−3^ corresponds to a plasma wavelength of *λ*_pe_ ≈ 1.1–3.3 mm. The uniformity of the vapour density is ensured by flowing a heat-exchanging fluid around a concentric tube surrounding the source at a temperature stabilized to ±0.05 °C. Longitudinal density differences of between −10% and +10% over 10 m may be implemented, and controlled at the 1% level. The motion of the (heavy) rubidium ions can be neglected during the transit of the proton bunch because they are singly ionized^35^.

### Witness electron beam

Production of the witness electron beam is initiated by illuminating a Cs_2_Te cathode by using a frequency-tripled laser pulse derived from the ionizing laser. Electron bunches with a charge of 656 ± 14 pC are produced and accelerated to an energy of 5.5 MeV in a 2.5 cell radio-frequency gun and are subsequently accelerated up to 18.84 ± 0.05 MeV using a 30 cell travelling wave structure. These electrons are then transported along an 18-m beam line before being injected into the vapour source. The focal point and crossing angle of the witness beam can be controlled via a combination of quadrupole and kicker magnets along this beam line.

### Background subtraction

The large distance between the camera and the proton beam line means that background noise generated by radiation directly incident on the CCD is minimal. The scintillator of the spectrometer, however, is subject to considerable background radiation. The rise and decay of the scintillator signal occur on timescales longer than 1 μs and, as such, the scintillator photons captured by the camera are produced by an indivisible combination of background radiation and accelerated electrons. The majority of this background radiation is due to the passage of the proton bunch and comes from two main sources: a 0.2-mm-thick aluminium window located 43 m upstream of the spectrometer between AWAKE and the SPS transfer line, and a 0.6-mm-thick aluminium iris at the downstream end of the vapour source. The inner radius of this iris is 5 mm, leading to negligible interaction with the standard SPS proton bunch. However, protons that are defocused during self-modulation, such as those measured at the downstream imaging station, can interact with the iris, creating a substantial background. The strength of the transverse fields in the plasma and hence the number of protons that are defocused is strongly dependent on the plasma density. Consequently, the background generated by the defocused protons is more substantial at higher plasma densities, such as the AWAKE baseline density of 7 × 10^14^ cm^−3^. At this density, the radiative flux on the scintillator due to the iris is much higher than that from the thin window. Conversely, at a lower plasma density, such as 2 × 10^14^ cm^−3^, the radiation from the iris disappears completely and the remaining incident radiation is produced almost entirely by the interaction of the protons with the upstream window.

Owing to the variable nature of the radiation incident on the scintillator, background subtraction is a multistep process. A background data sample with the electron beam off at a plasma density of 1.8 × 10^14^ cm^−3^ is taken, such that the background has two key components: one due to the camera readout and ambient light in the experimental area, and another, *N*_p_-dependent background caused by the proton bunch passing through the thin window. For each pixel imaging the scintillator, a linear function of *N*_p_ is defined by a *χ*^2^ minimization fit to the background data sample, giving an *N*_p_-dependent mean background image. For each signal event, a region of the scintillator is chosen where no accelerated electrons are expected, typically the lowest-energy part, and the background is rescaled by the ratio of the sums over this region in the signal event and the *N*_p_-scaled background image. At higher plasma densities, a further step is needed to subtract the background from the iris. This background falls rapidly with increasing distance from the beam line and therefore depends on the horizontal position in the plane of the scintillator. A new region where the expected number of accelerated electrons is small is chosen, this time along the top and bottom edges of the scintillator. The mean of each column of pixels in this region is calculated and then subtracted from each pixel in the central region of that same column, leaving only the signal. The semi-circular ends of the scintillator reduce the effectiveness of this technique at the highest and lowest energies.

### Signal extraction

To obtain an accurate estimate of the electron-bunch charge, the background-subtracted signal is corrected for two effects that vary across the horizontal plane of the scintillator. One effect comes from the variation in the horizontal angle of incidence of the electron on the scintillator. This angle is determined by the same tracking simulation used to define the position–energy relationship, and introduces a cosine correction to the signal owing to the variation in the path length of the electron through the scintillator. The second effect is vignetting, which occurs as result of the finite size of the optics of the spectrometer and the angular emission profile of the scintillator photons. A lamp that mimics this emission profile is scanned across the horizontal plane of the scintillator and the vignetting correction is determined by measuring its relative brightness. The increase in radiation accompanying the electron bunch, owing to its longer path length through the vacuum window at larger incident angles, is negligible and therefore does not require an additional correction factor.

### Data reporting

No statistical methods were used to predetermine sample size.

## Online content

Any methods, additional references, Nature Research reporting summaries, source data, statements of data availability and associated accession codes are available at 10.1038/s41586-018-0485-4.

## Data Availability

The datasets generated and analysed during this study are available from the corresponding author on reasonable request. The software code used in the analysis and to produce Figs. 2–4 is available from the corresponding author on reasonable request.
